# Patterns, Associated Factors, and Clinical Outcomes of Poisoning among Poisoning Cases Presented to Selected Hospitals in Western Ethiopia: Hospital-Based Study

**DOI:** 10.1155/2020/5741692

**Published:** 2020-05-06

**Authors:** Ashenafi Habte Woyessa, Thanasekaran Palanichamy

**Affiliations:** ^1^Wollega University, College of Health Science, School of Nursing and Midwifery, Department of Emergency and Critical Care Nursing, Nekemte, Ethiopia; ^2^Wollega University, College of Health Science, School of Nursing and Midwifery, Department of Psychiatric Nursing, Nekemte, Ethiopia

## Abstract

**Results:**

The broad types of poisoning were identified in about 193 (91.47%) cases of poisoning in this study. Pesticides exposure and food poisoning have, respectively, contributed for 32.70% and 20.91% of the poisoning incidence. On the other hand, chemical from industry has contributed the least percentage (2.81%). Out of a total of 24 agents identified, 26.80% of the agents were organophosphates followed by raw meat (18.40%). Difference in the incidence of poisoning was also observed as seasons in a year change. Among the victims who have taken household materials as a poisoning agent, about 47.87% of them have taken the agents during daytime. The remaining cases of poisoning developed by household chemicals occurred at night. More than half (54.98%) of the poisoned patients have encountered the incidents inside their home. Regarding the final poisoning outcome, about 7.10% poisoning cases in this study died of the poisonings. Factors such as place, time, intention, and source of poisoning were observed to determine poisoning outcomes. Although poisoning attempt was lesser among urban residents as compared to rural community, rural dwellers were four times more likely to die of poisoning they had attempted (AOR: 4.072 (1.197–13.85)).

**Conclusion:**

This study has clearly showed that the incidence of poisoning was varied with seasonal variations. The encountered poisonings ended up with mixed clinical outcomes, which were also affected by patients' demographic and clinical characteristics. Fertilizers, unclean food items, household materials, and drugs have caused majority of the poisonings. Creating community awareness and designing sound prevention strategies are recommended to reduce morbidity and mortality related to poisoning.

## 1. Introduction

Poisoning is one of the major health and health-related problems of our nations. It causes significant mortality and morbidity in many countries. In the underdeveloped countries, the increasing incidence of poisoning is exceptionally resulting in significant threats including hospitalization and huge financial crisis. According to a recent report by the World Health Organization, many thousands of people worldwide lose their life annually due to unintentional poisoning, of which more than 90% of the deaths occur in the developing countries [1].

Both pattern and magnitude of poisoning in a particular place rely on various contributing factors. Nature of poisoning usually varies across the world. Due to variation in the socioeconomic factors and cultural diversity, the distribution of poisoning is not uniform even in different parts of the same country. Magnitude of poisoning in a particular area is determined by availability of poisonous agents, the dominant occupation in that society, and religious and sociocultural influences [2, 3]. More importantly, in the underdeveloped countries, unsatisfactory drug and chemical regulation, inadequacy of surveillance systems, poor enforcement, and easy access to various kinds of drugs or chemicals have been accused for the higher poisoning prevalence [4, 5].

Rapid development of industry in wealthy nations and massive utilization of pesticides in developing countries to increase agricultural products have increased the incidence of poisoning. Similarly, with the advances in medical sciences, a vast number of insecticides and drugs have become available, which when exposed to them could end up with severe toxicity [3, 6, 7]. Furthermore, expansions of technology and social development have contributed in the availability of many other dangerous chemical substances in contemporary community [8, 9].

Absence of facilities for safe storage and disposal are additional contributors for commonly encountering accidental poisonings, particularly among small children. Developmental issues and peer pressures also contribute to the increased vulnerability of poisoning among young adults. The goal of independence and tendency to believe that they are everlasting may put adolescents at undue risks in experimentation with different substance abuse including illicit drugs [10].

Ethiopia, one of the low income sub-Saharan African countries, shares its considerable burden of poisoning emergencies. One retrospective study in Ethiopia found 8.6% fatality rate among followed cases [11]. Since agriculture is the main occupation in the country, insecticides are used to a greater extent and poisonings with such products are becoming very common. An elucidative and recent study undertaken in Addis Ababa, capital of Ethiopia, reveals that the leading cause of poisoning was organophosphate (43.1%) and most deaths were also caused by this specific agricultural input pesticide [12].

However, the pattern and main risks of poisoning accidents change with time and place. As such, epidemiological surveillance specific to each geographical location is necessary to determine the extent and characteristics of the problem, according to which related preventive measures can be taken. Furthermore, availability of information regarding poisoning outcome beyond prevalence is instrumental to uncover the significance of the problem and helps excel the efforts to curtail the problem [13].

Availability of information on types, characteristics, and extent of poisoning in a specific region is important not only for diagnosis and management purpose but also to introduce preventive measures for future [7]. Nevertheless, in Ethiopia, the epidemiological data and overall information regarding the significance of poisoning and its specific clinical outcomes are unknown. The limited studies undertaken in other regions of the country have not assessed the pattern and clinical outcomes of the reported poisoning.

This study was therefore carried out to assess the patterns, associated factors, and clinical outcomes of poisoning among poisoned patients presented to four selected hospitals in western Ethiopia. Information of the patterns in terms of nature, severity, and outcome could be helpful in the design of interventions geared at the reduction of morbidity and mortality by taking up appropriate planning, prevention, and management techniques.

## 2. Materials and Methods

### 2.1. Study Setting and Period

This study was conducted in four hospitals selected from three administrative zones of Wollega in the western part of Ethiopia. The study has been undertaken for 11 months from January 10, 2018, to November 10, 2018.

### 2.2. Study Design

Prospective cross-sectional study design was employed to assess patterns, associated factors, and clinical outcomes of poisoning among poisoned patients presented to the selected hospitals.

### 2.3. Sampling Procedure and Techniques

Area sampling technique was used to select the four hospitals. Initially, five administrative zones, namely, Buno, Kelem Wollega, West Wollega, East Wollega, and Horo Guduru Wollega, were selected as geographical clusters from western Ethiopia. Out of them, three zones (West Wollega, East Wollega, and Horo Guduru Wollega) were randomly selected. Then, four hospitals (Nekemte, Shambu, Gimbi, and Nejo hospitals) were purposively selected. Since the objective of the study was to determine the pattern and outcome of poisoning during the specified study period, no specific sampling size determination was employed. As such, a total of 211 poisoned cases who presented to the selected hospitals during the mentioned study period were consecutively included.

### 2.4. Research Tool and Data Collection Techniques

Data were collected using a comprehensively organized and pretested interviewer-administered questionnaire, which was developed after reviewing updated literatures. The questionnaire comprises of four parts (sociodemographic characteristics and associated factors, base line information, pattern, and outcomes of poisoning). A total of eight data collectors (two for each hospital) were recruited. In order to familiarize them with collection of the required data and upholding the confidentiality, training was given by investigators.

### 2.5. Data Processing and Analysis

Data were initially entered into epi-data version 3.2 by data clerks. After several steps of check for completeness and accuracy, the data were then exported to SPSS program version 20 for analysis. Pattern and outcomes of poisoning cases were described by sociodemographic and clinical characteristics. Possible association among independent variables and outcome variables was examined using odds ratio and 95% confidence interval as a measure of association. Finally, the results of the study were presented using text, tables, charts, and graphs.

## 3. Results

This research is an epidemiological study of poisoning cases that aimed to assess the seasonal pattern, associated factors, and clinical outcomes of poisoning cases presented to selected hospitals in western Ethiopia. The key findings of the study related to sociodemographic characteristics, base line information of respondents, seasonal patterns of poisoning incidence, characteristics of poisoning, and clinical outcomes with their contributing factors are presented in subsequent sections.

### 3.1. Sociodemographic Characteristics and Base Line Information of Respondents

In the present study, the highest proportion (45.97%) of the poisoning cases was in the age category of 16–30 years. About two-thirds (59.20%) of poisoned cases were female. Regarding their marital status, more than half (55%) were single. Regarding living condition of the victims, 56.40% of them have reported that they were living with their family in rural areas. The highest monthly income of about 32.23% respondents was between 500 and 750 Ethiopian birr. As shown in this study, 32.70% of the poisoning cases have history of previous suicidal attempt among which 63.77% have got counseling services (Tables 1 and 2).

### 3.2. Seasonal Pattern of Poisoning Cases at the Study Area

**Figure 1:**
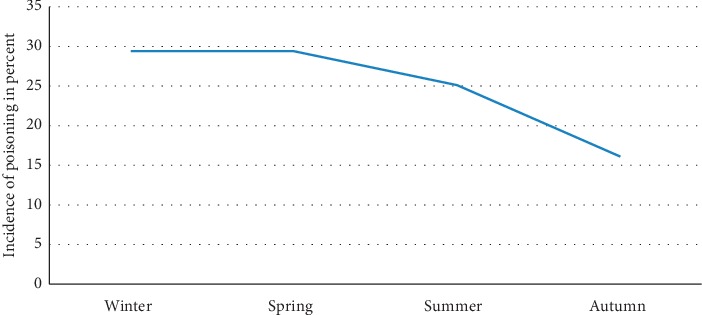
Seasonal patterns of poisoning in Nekemte, western Ethiopia, 2018. Key: autumn = September to November; winter = December to February; spring = March to May; summer = June to August.

This study has assessed the patterns of poisoning incidences in terms of the four seasons of the year according to the Ethiopian calendar. Relatively largest proportions (30%) of the admitted cases were in the months from December to February. The admission due to poisoning at the area has gradually decreased to its least record in the months from September to November. From this finding, it can be clearly seen that the incidence of the problem gradually decreases from winter to autumn (Figure 1).

### 3.3. Poisoning Types, Clinical Outcome, and Contributing Factors

**Figure 2:**
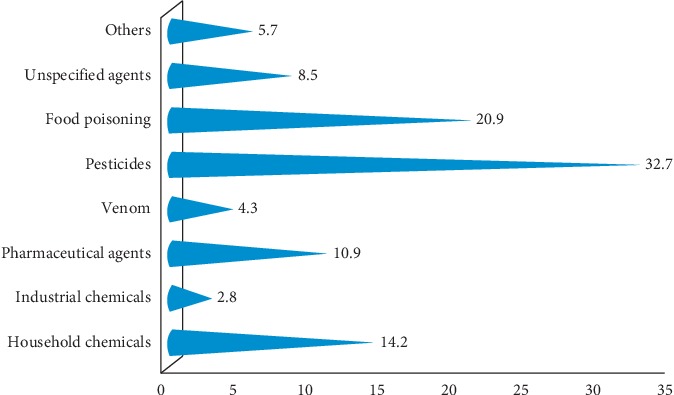
Distribution of poisoning cases by their types of poisoning agents in western Ethiopia, 2018.

In about 193 (91.47%) of poisoning cases in this study, the broad types of poisoning were identified. In the remaining 8.53% of cases, the types of poisoning were not identified. In the group of victims, physicians have used few favoring symptoms to diagnose as having poisoning. Out of 193 people in whom the causes of poisoning were identified, pesticides and food poisoning have, respectively, contributed to 32.70% and 20.91% of the incidence. Chemical from industry has contributed the least percentage (2.81%). The types of poisoning are categorized as “others” which include causes such as alcohol and “unspecified” mean poisoning, which occurred by unknown underlying causes (Figure 2).

On the other hand, this study has also identified specific poisoning agents with which the patients were poisoned. Out of a total of 24 agents identified, 26.80% were organophosphate that was followed by raw meat (18.40%) that is the commonly eaten type of meat in Ethiopia (Table 3).

### 3.4. Clinical Symptoms, Measures Taken, and Complications Developed

While generalized abdominal pain was reported by 28.91% of the study participants, unconsciousness (19.43%), change in skin color (16.59%), and shortness of breath (3.79%) were also among the observed clinical presentations. Majority (79.60%) of poisoning cases presented to the selected hospitals have not received prehospital care. Regarding remedies given in the hospitals, 68.72% of the victims have received general resuscitation with specific supervision. No complications have been reported in about 57.35% of the poisoned patients presented to the selected hospital during this particular study (Table 4).

### 3.5. Poisoning Outcomes and Common and Contributing Factors

Out of 211 poisoning cases, 196 (92.90%) survived and the remaining 15 (7.10%) died of poisoning they have encountered. Distribution of poisoning incidence in terms of place, time, intention, and source of poisoning was among the selected variables to determine poisoning outcomes. Among persons who took household materials as poisoning agents, 47.87% have taken during daytime. Poisoning occurred at night time in about 31.28% of cases. Regarding place of poisoning, more than half (54.98%) have exposed to the agents inside their homes (Table 5).

This study found that the poisoning attempt of rural respondents was 58.70%, and their death rate was 26.70%. The attempt of poisoning of the urban population is less compared to rural (58.70% for rural and 41.30% for urban dwellers). However, demise was four times likely in the urban population as compared to the victims who were rural residents (AOR: 4.072(1.197–13.85); *P* value: 0.02) (Table 6).

## 4. Discussion

This study has investigated poisoning in relation to the seasonal pattern of incidence, associated factors, and clinical outcomes among poisoning cases who have visited the selected public hospitals in western Ethiopia. Key findings of the study are compared and contrasted with the existing literatures and presented briefly.

Poisoning is one of the challenging public health problems that are causing significant morbidity and mortality throughout the globe. Its incidences are increasing from day to day. As a result of change in lifestyle and social behavior of the contemporary society, in all aspects burden of poisoning is increasing more than ever before. As revealed in many literatures, distress due to loss of business, failure in romance or differences with the intimate partner or examination, emotional disturbances, and chronic diseases are the common reasons for intentional poisoning. Acute pesticide poisoning is one of the most common causes of intentional deaths worldwide [14].

In general, the rapid and extensive development of technological products and social development have paved the way for easy availability of most harmful chemicals substances in our community. In less industrialized setting, pesticide chemicals and medicinal drugs are among the notorious agents causing the poisoning. Regardless of intentional or accidental exposure, it is the easy access to these substances that significantly adds the incidence. Toxicity as a result of drugs and chemicals is greatly influenced by the population's socioeconomic and cultural differences [15].

Seasonal disparity of poisoning incidences was observed during the specific study period of this particular study. The months from December to February were found to be a point in time when maximum poisoning cases were admitted. On the other hand, the incidence was observed to steadily decrease for other seasons up to its lowest record in the months from September to November. Studies undertaken in Palestine and elsewhere have also revealed similar seasonal fluctuations in the actual incidences of poisoning occurrences [16, 17].

The socioeconomic, culture, and other demographic characteristics are among the factors that either positively or negatively sway the poisoning incidence. In this study, the age of poisoning cases was mostly in the range of 15–30 years. About two-thirds (59.20%) of poisoning victims were female. Regarding marital status, more than half (55%) of the poisoned victims were unmarried. As to the living condition of patients at the time of admission to the hospitals, 74.41% of respondents reported that they were living with their family in rural areas. A prospective study undertaken at Sher-i-Kashmir Institute of Medical Sciences found that higher incidence of poisoning was observed among young females (age group of 12–24 years) of rural areas [18].

Plenty of studies have proved that chemicals that serve as pesticides are the commonest cause of poisoning in the vast majority of less industrialized countries where agriculture is overriding their economy. Beyond ready availability of highly toxic pesticides, poor culture of safety which is contributed by illiteracy, ignorance, and lack of protective devices were the main reasons of exposure in these setting. In our study, poisoning caused by pesticides accounts for the higher percentage (32.7%) followed by food poisoning (20.9%). But this finding is different from the finding of a similar study carried out in Addis Ababa, which found household cleaning agents as the leading cause of poisoning (43.1%) followed by organophosphate (21.6%) [13, 15]. The observed discrepancy might be due to the fact that Addis Ababa, the capital of Ethiopia, is more industrialized than our study area where chemicals from industry were more likely to cause poisoning.

In the present study, incidence of poisoning in nearly half (46.45%) and 35.54% of the victims was, respectively, intentional and accidental. A survey conducted in India to assess the prevalence and mortality incidence rate due to various poisoning agents found that 68.40% of cases were due to intentional poisoning and 31.60% were due to accidental poisoning [19]. The observed variation between the two studies might be due the difference of socioeconomic status between the two countries, which might also be related to availability and accessibility of poisoning agents.

The treatment assessment result showed that appropriateness with the specific treatment was 92.90%. The remaining 7.10% cases did not receive the specific appropriate treatment. Among all victims, only 43 (20.40%) cases received prehospital care. The finding of treatment appropriateness in this study is better when compared with the findings of a similar study performed to look at the treatment assessment, which found only appropriate treatment in 78.7% of clients [20]. The difference between the two findings might be due to progressive improvement in the assessment and treatment of poisoning cases.

In our study, the mortality rate due to poisoning was 7.10%, and this was mostly among patients who presented with severe symptoms and presented lately. This is nearly a similar finding with that in an Addis Ababa study which found 8.6% of death from poisoning but bigger finding as compared to a finding from Gondor, Ethiopia which found only 2.4% poisoning death. Nevertheless, studies conducted in other countries including Zimbabwe generally showed a higher case fatality ratio [21, 22].

Poisoning attempt and death rate could be affected by sociodemographic factors of the study participants. While rural attempt was bigger compared to the percentage of attempt by town dwellers (48.7% rural attempt versus 41.3% urban attempt), urban death from poisoning was much bigger (73.3%) than death in rural dwellers. This is not in line with findings reported from many other countries [23, 24]. The discrepancy might be due to variation in quality of emergency and critical care services provided by respective hospitals in various countries.

Generally, the magnitude, characteristics, and the clinical outcomes of poisoning are largely determined by individual sociodemographic variables, the dominant occupation in a society, commonly available and accessible type of poisoning agents, specific point in time, and setting of the poisoning incidents [2–5].

## 5. Conclusion

This study has clearly showed that there has been seasonal variation in incidences of poisoning cases, which were also ended up with mixed clinical outcomes. The outcomes of patients were found to vary with patients' demographic and clinical characteristics. Mishandling of fertilizers in agricultural areas, eating contaminated food items, and easy accessibility of household chemicals and medicines have caused majority of the poisoning in this study area. Therefore, generating community awareness and designing sound prevention strategies must be considered to reduce morbidity and mortality related to poisoning.

## 6. Strengths and Limitations of the Study

### 6.1. Limitations

As the findings are obtained from health facilities alone, the results may not be the true reflection of the problem of the general population. In addition, as patients were followed up only during their stay at the hospitals, the study did not reflect the fate of referred cases and those who were discharged against medical advice.

### 6.2. Strengths

The prospective nature of the study design enabled the researchers to deal with the actual spectrum and nature of poisoning incidences and their outcomes which might otherwise be impossible to achieve using retrospective type.

## Figures and Tables

**Table 1:** Sociodemographic characteristics of the study participants in western Ethiopia, 2018.

No	Sociodemographic characteristics(*N* = 211)	Category	Frequency	Percent
1	Age of the patient	<5 years	30	14.22
		6–15 years	44	20.85
		16–30 years	97	45.97
		31–45 years	21	9.95
		46–60 years	12	5.69
		+61 years	7	3.32

2	Sex	Male	86	40.80
		Female	125	59.20

3	Ethnicity	Oromo	154	72.99
		Amhara	17	8.057
		Tigre	17	8.057
		Gurage	14	6.635
		Others	9	4.265

4	Marital status	Single	116	54.98
		Married	73	34.60
		Divorced	12	5.69
		Widowed	6	2.84
		Cohabitated	4	1.89

5	Level of education attained	No schooling	36	17.06
		Primary school	79	37.44
		Secondary school	77	36.49
		Higher institution	19	9.00

6	Monthly income	150–500	48	22.75
		501–750	68	32.23
		751–1500	31	14.69
		1501–3000	15	7.10
		3001–5000	8	3.79
		+5001	7	3.32
		Not applicable (due to age)	34	16.11

7	Religion	Orthodox	42	19.91
		Protestant	102	48.34
		Catholic	41	19.43
		Muslim	18	8.53
		Others	8	3.79

8	Residence	Town	92	43.60
		Rural	119	56.40

9	Occupation	Government employee	13	6.16
		Nursery	8	3.79
		Maid/servant	10	4.74
		Housewife	28	13.27
		Merchant	24	11.37
		Farmer	43	20.38
		Laborer	19	9.00
		Student	49	23.22
		Driver	7	3.32
		Others	10	4.74

**Table 2:** Base line information of the study participants in western Ethiopia, 2018.

No	Variables	Response	Frequency	Percent
1	Living condition	Alone in the house	37	17.54
		Alone on the street	11	5.21
		With family/friends	157	74.41
		Others	6	2.84

2	Presence of comorbidity	Yes	42	19.91
		No	149	70.62
		Unknown	20	9.47

3	Body system involved by comorbidity (*N* = 42)	Neurologic system	6	14.29
		Endocrine system	11	26.19
		Cardiovascular system	9	21.43
		Renal system	9	21.43
		Gastrointestinal system	3	7.14
		Others	4	9.52

4	History of psychiatric disorder	Yes	23	10.90
		No	188	89.10

5	Previous history of suicide attempt	Yes	69	32.70
		No	119	56.40
		Unknown	23	10.90

6	Counseling received(*N* = 69)	Yes	44	63.77
		No	25	36.23

**Table 3:** Distribution of poisoning cases by specific poisoning agents in western Ethiopia, 2018.

Specific poisoning agents (*N* = 175)	Frequency	Percent
Kerosene(carbon monoxide)	4	2.23
Disinfectant	5	2.79
Rat killer	8	4.47
Ant killer	3	1.68
Mosquito repellant	5	2.79
Food additive	3	1.68
Snake	3	1.68
Bee	1	0.56
Wasp	1	0.56
Scorpion	1	0.56
Rabid dog	2	1.12
Organophosphate	48	26.80
Insecticide	11	6.15
Rodenticide	10	5.59
Analgesics	4	2.23
Anti-acids	1	0.56
Antibiotics	8	4.47
Anticonvulsants	2	1.12
Antihypertensive	1	0.56
Antimalarial	1	0.56
Antiretroviral	1	0.56
Raw meat	33	18.40
Dairy products	7	3.91
Alcohol	12	6.70

**Table 4:** Symptoms, treatments provided, and complications developed in Nekemte, Ethiopia, 2018.

No	Clinical symptoms (*N* = 211)	Category	Frequency	Percent
1	Presenting chief compliants	Headache	9	4.27
		Skin color change	35	16.59
		Disorientation/unconsciousness	41	19.43
		Vomiting/diarrhea	41	19.43
		Epigastria/abdominal pain	61	28.91
		Shortness of breath	8	3.79
		Unknown	16	7.58

2	Measures taken	Prehospital care	43	20.40
		General resuscitation	145	68.72
		Specific antidote administration	51	24.17
		Other measures	15	7.11

3	Complications developed	Developed	78	36.97
		Not developed	121	57.35
		Not identified	12	5.68

**Table 5:** Common contributing factors associated with poisoning among poisoned patients in western Ethiopia, 2018.

No	Base line information(*N* = 211)	Category	Frequency	Percent
1	Place of poisoning	Home	116	54.98
		Work place	74	35.07
		Unknown	21	9.95

2	Time of poisoning	Day time	101	47.87
		Night time	66	31.28
		Weekend time	44	20.85

3	Source of poison	Household materials	98	46.45
		Shop	66	31.28
		Pharmacy	24	11.37
		Others	23	10.90

4	Intention of poisoning	Accidental	75	35.54
		Deliberate self-harm	98	46.45
		Recreational	25	11.85
		Unknown	13	6.16

5	Any home remedy given?	Yes	81	38.39
		No	119	56.40
		Unknown	11	5.21

6	Mode of hospital arrival	People shoulder	28	13.27
		Public car	89	42.18
		Public ambulance	61	28.91
		On foot	33	15.64

7	Time elapsed since exposure to arrival at hospital	<2 hours	43	20.38
		2–4 hours	101	47.87
		>4 hours	67	31.75

8	Root of poisoning	Ingestion	148	70.14
		Direct contact	37	17.54
		Inhalation	8	3.79
		Injection	10	4.74
		Unknown	8	3.79

9	Frequency of exposure to toxins	One	136	64.46
		Two	46	21.80
		Three	7	3.32
		Four times and above	22	10.42

**Table 6:** Association of poisoning outcome with selected sociodemographic variables (*n* = 211) in western Ethiopia, 2018.

Variables	Category	Survival	Death	OR	CI (95%)	*P* value	AOR	CI (95%)	*P* value
Level of education attained	No schooling	34 (17.3%)	2 (13.3%)	0.314	0.048–2.067	0.228	0.358	0.051–2.511	0.302
	Primary school	75 (38.3%)	4 (26.7%)	0.284	0.058–1.397	0.122	0.337	0.064–1.763	0.198
	Secondary school	71 (36.2%)	6 (40.0)	0.451	0.102–1.996	0.294	0.467	0.098–2.234	0.340
	Higher institution	16 (8.2%)	3 (20.0)	1 (R)	1 (R)	1 (R)	1 (R)	1 (R)	1 (R)

Residence	Urban	81 (41.3%)	11 (73.3)	0.256	0.079–0.833	0.024	4.072	1.197–13.85	0.02^*∗∗*^
	Rural	115 (58.7%)	4 (26.7%)	1 (R)	1 (R)	1 (R)	1 (R)	1 (R)	1 (R)

Occupation	Government employee	12 (6.1%)	1 (6.7%)	3.500	0.203–60.210	0.388	2.965	0.166–53.098	0.460
	Private employee	97 (49.5%)	9 (60.0%)	3.897	0.478–31.742	0.204	3.533	0.416–29.98	0.247
	Student	45 (23.0%)	4 (26.7%)	3.733	0.401–34.764	0.247	2.626	0.266–25.892	0.408
	Farmer	42 (21.4%)	1 (6.7%)	1 (R)	1 (R)	1 (R)	1 (R)	1 (R)	1(R)

## Data Availability

The raw data supporting our findings are available from the authors upon a reasonable request.
